# Deciphering the Role of Residues Involved in Disorder-To-Order Transition Regions in Archaeal tRNA Methyltransferase 5

**DOI:** 10.3390/genes12030399

**Published:** 2021-03-11

**Authors:** Ambuj Srivastava, Dhanusha Yesudhas, Shandar Ahmad, M. Michael Gromiha

**Affiliations:** 1 Department of Biotechnology, Bhupat and Jyoti Mehta School of Biosciences, Indian Institute of Technology Madras, Chennai 600036, India; ambuj.88.in@gmail.com (A.S.); dhanusha2504@gmail.com (D.Y.); 2 School of Computational and Integrative Sciences, Jawaharlal Nehru University, New Delhi 110067, India; shandar@jnu.ac.in

**Keywords:** tRNA methyltransferase, protein-RNA complexes, Trm5/aTrm5, disordered regions, disorder-to-order transition, molecular dynamics simulations

## Abstract

tRNA methyltransferase 5 (Trm5) enzyme is an S-adenosyl methionine (AdoMet)-dependent methyltransferase which methylates the G37 nucleotide at the N^1^ atom of the tRNA. The free form of Trm5 enzyme has three intrinsically disordered regions, which are highly flexible and lack stable three-dimensional structures. These regions gain ordered structures upon the complex formation with tRNA, also called disorder-to-order transition (DOT) regions. In this study, we performed molecular dynamics (MD) simulations of archaeal Trm5 in free and complex forms and observed that the DOT residues are highly flexible in free proteins and become stable in complex structures. The energetic contributions show that DOT residues are important for stabilising the complex. The DOT1 and DOT2 are mainly observed to be important for stabilising the complex, while DOT3 is present near the active site to coordinate the interactions between methyl-donating ligands and G37 nucleotides. In addition, mutational studies on the Trm5 complex showed that the wild type is more stable than the G37A tRNA mutant complex. The loss of productive interactions upon G37A mutation drives the AdoMet ligand away from the 37th nucleotide, and Arg145 in DOT3 plays a crucial role in stabilising the ligand, as well as the G37 nucleotide, in the wild-type complex. Further, the overall energetic contribution calculated using MMPBSA corroborates that the wild-type complex has a better affinity between Trm5 and tRNA. Overall, our study reveals that targeting DOT regions for binding could improve the inhibition of Trm5.

## 1. Introduction

The biosynthetic pathway of tRNA requires several post-transcriptional modifications in nucleotides, such as the isopentylation, methylation, capping, and conversion of uridine to dihydro-uridine, thio-uridine, or pseudo-uridine, and methylation is the most frequent among them [1]. These tRNAs require an L-shaped structure for the efficient translation process, and the modification at the 37th position is shown to be essential for the stabilisation of the functional L-shape and reading frame maintenance [2,3,4]. tRNA methyltransferase (Trm5) proteins are mainly involved in the methylation of many tRNAs from all domains of life. Specifically, Trm5 methylates the guanine nucleotide at the 37th position in archaea (aTrm5) and eukaryotes (eTrm5) [5,6,7]. This modification is present in many tRNAs, including tRNA^Leu^, tRNA^Pro^, tRNA^His^, tRNA^Gln^, and tRNA^Arg^, and is likely to be conserved in all three domains of life (bacteria, archaea, and eukarya) [8,9]. The Trm5 methylates the G37 nucleotide using S-adenosyl methionine (AdoMet) as a donor of the methyl group [10]. Various studies have shown that these modifications play a major role in increasing the accuracy and efficiency of the protein translation process [11,12,13,14]. Further, methylations are shown to be involved in RNA quality control systems and the regulation of tRNA localisation in the cell. The archaea and eukaryotes have Trm5 enzyme, while the bacteria have tRNA methyltransferase D (TrmD) for the methylation of G37 [15,16,17].

tRNA modification enzymes can be categorised into multiple classes. Trm5 and TrmD enzymes methylate at the N^1^ atom of G37 and belong to class I and IV, respectively [15,17,18]. Class I enzymes are L-shape dependent, while class IV enzymes do not require an L-shape to perform their functions. Moreover, class I and class IV enzymes are monomeric and dimeric, respectively, in stoichiometry [17].

Protein-tRNA complexes often exhibit intrinsically disordered regions (IDRs), which are highly flexible and lack stable three-dimensional structures [19]. These IDRs attain ordered structures upon binding with their partners, a process known as disorder-to-order transition (DOT), and correspondingly the regions undergoing these transitions are called DOT regions [20]. Many protein-tRNA complexes, such as tRNA synthetases, tRNA modification enzymes, and tRNA pre-processing enzymes, contain IDR and DOT regions [21]. Particularly, the tRNA modification enzyme TrmD forms a homodimeric structure for the methylation of tRNA which also contains a disordered region present in the interdomain region of the N and C terminal domains [17,22]. Ahn et al. [22] showed that the disordered regions are likely to be involved in the methylation process. The archaeal counterpart of TrmD—i.e., aTrm5—also contains disordered regions in the free proteins, which attain an ordered structure upon binding with tRNA.

The IDR and DOT regions are crucial for the folding of several protein complexes. Recent kinetic studies have shown that the binding and folding of intrinsically disordered proteins is a coupled process [23,24]. Theoretically, disordered regions are solvent-exposed loops which change their conformations frequently and attain a stable structure upon binding [20]. Hence, a stable folding of protein complexes is expected to be observed after binding with their partners. In this work, we analysed aTrm5 enzyme from *Methanocaldoccocus jannaschii*, which contains three DOT regions: DOT1 (69–75), DOT2 (81–90), and DOT3 (138–145). Goto-Ito et al. [5,25] solved the structure of free and bound forms of aTrm5 and showed that residues 137–147 in the DOT3 region are disordered and attained ordered structures upon binding with tRNA, whereas discussions on DOT1 and DOT2 were missing in their work [5,25]. We performed a detailed analysis of all DOT regions and their involvement in interactions with tRNA, as well as the mechanism of action for tRNA methyltransferase protein. In addition, we discussed the role of these DOT regions for binding with tRNA, especially the influence of DOT3 in transferring the methyl group on the G37 nucleotide. Further, we investigated the effect of G37A mutation in the tRNA towards the interactions with aTrm5.

We found that the DOT regions are less flexible in the presence of tRNA, while the corresponding disordered residues in the free protein are highly flexible. However, the overall structure is compact in the free protein due to intramolecular interactions throughout the simulations. The structural analysis and molecular dynamics simulation studies suggest that DOT regions are crucial for maintaining compact structure of the free protein, as well as stabilising the protein-tRNA complex. An analysis of specific positions on tRNA suggests that the G37 nucleotide is known to be very important, and the mutation G37A is energetically unfavourable.

## 2. Methods and Materials

### 2.1. Dataset

The crystal structure of aTrm5 complex (PDB code: 2ZZM) [25] and the free protein (PDB code: 2YX1) [5] with 2.65 Å and 2.20 Å resolution, respectively, were downloaded from the Protein Data Bank [26]. The annotation of disordered regions was obtained from the missing coordinate information given in the “REMARK 465” section of the PDB file. We obtained the DOT regions by comparing the regions which are missing in the free protein, and having coordinate information in the aTrm5 complex [19]. In aTrm5 protein, we observed three DOT regions—DOT1 (69-KIIKKPS-75), DOT2 (81-SKKYRKEIDE-90), and DOT3 (138-SEVKGEFR-145)—which were modelled using the program Modeller 2.19 [27]. Subsequently, we generated 10 models and selected the structure that had the lowest DOPE score [28]. Further, we mutated guanine at position 37 to adenine (G37A) of the tRNA using the Chimera program and performed MD simulations to study the importance of the G37 nucleotide of the tRNA in the aTrm5 complex.

### 2.2. Molecular Dynamics Simulations

We performed molecular dynamics simulations of aTrm5 complex for 100 ns using the AMBER12 program and the FF12SB force field [29]. The TIP3PBOX water model was used to solvate the molecule with the edge of the box at least 10 Å away from solute molecules [30]. Na^+^ ions were added to neutralise the molecule, since the net charge was negative. The energy minimisation of each molecule was carried out for 50,000 steps.

To study disorder-to-order transition, we simulated the protein-tRNA complex, unliganded complex, and the free protein, as well as G37A tRNA mutant in the protein-tRNA complex. The simulations of wild-type and G37A tRNA mutant complexes were repeated with a random seed to examine the consistency of the results. To explore the stabilised and equilibrated structures, we analysed the last 80 ns of the simulations.

### 2.3. Trajectory Analysis

Trajectory visualisation and analysis were performed using PYMOL 1.8.4, Chimera, and VMD [31,32]. Various CPPTRAJ commands were used for the trajectory analysis. The residue movements—i.e., the root mean square fluctuation (RMSF)—were calculated using the “atomicfluct” command of the CPPTRAJ program. Minimum distance calculations were performed using “nativecontacts”, and the radius of gyration was computed using “radgyr”, which is available in the CPPTRAJ program [33].

The binding free energy calculations of wild-type and aTrm5 mutant tRNA complexes were performed using MMGBSA and MMPBSA methods with an ionic strength of 0.1 M [34]. The last 10 ns trajectories were considered for the calculation, using tRNA as a ligand and protein as the receptor. The binding free energy was computed using Equation (1). In addition, we calculated the decomposition of energy per residue. The energetic calculations involve multiple components such as van der Waals (E_vdW_), electrostatics (∆E_ele_), polar (∆G_polar_), and nonpolar (∆G_nonpolar_), as presented in Equation (2).

(1) ∆Gbind = GPL − GP − GL,

(2) ∆G = ∆EvdW + ∆Eele + ∆Gpolar + ∆Gnonpolar.

## 3. Results and Discussion

### 3.1. Role of DOT Regions

**Figure 1:**
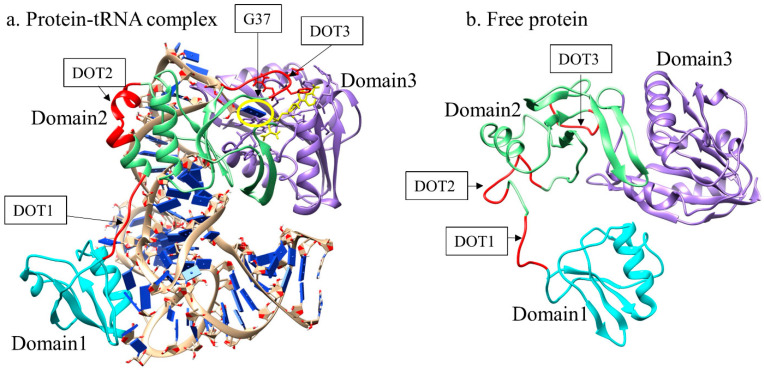
Archaeal tRNA methyltransferase enzyme in (**a**) complex form and (**b**) free protein. The domain 1 (D1), domain 2 (D2), and domain 3 (D3) are shown in cyan, green, and purple colours, respectively. The disorder-to-order transition (DOT) regions are highlighted with red colour, and G37 nucleotide is shown in the yellow oval.

The aTrm5 proteins are divided into three domains—namely, domain1 (D1: 1–68), domain2 (D2: 76–175), and domain3 (D3: 176–336) [5]. The DOT1 is present at the junction of domain1 (D1) and domain2 (D2), while the other two DOT regions are present in domain2 of the complex (Figure 1). tRNA is a large molecule, and aTrm5 enzyme is wrapped around the tRNA molecule with the help of flexible DOT regions. These regions showed an extended conformation in the complex, presumably because it allows a better binding of the protein and tRNA. The MD simulations of the complex showed that most of the DOT residues bind with the tRNA; in the free protein, these residues provide essential flexibility to facilitate a compact shape (Figure 1). DOT1 and DOT2 are mainly observed to be important for the interactions with tRNA, whereas DOT3 region binds to the G37 nucleotide, as well as S-adenosyl methionine (AdoMet) ligand, and stabilises the complex. The distances of DOT regions from G37 nucleotide and AdoMet ligand are shown in Figure S1a,b, respectively. The results showed that both AdoMet ligand and G37 are close to DOT3 regions with a distance of about 2.5 Å throughout the simulations, while DOT1 and DOT2 are far away from AdoMet ligand and G37 nucleotide (25 Å and 15 Å, respectively).

#### 3.1.1. Residue Fluctuation Analysis

**Figure 2:**
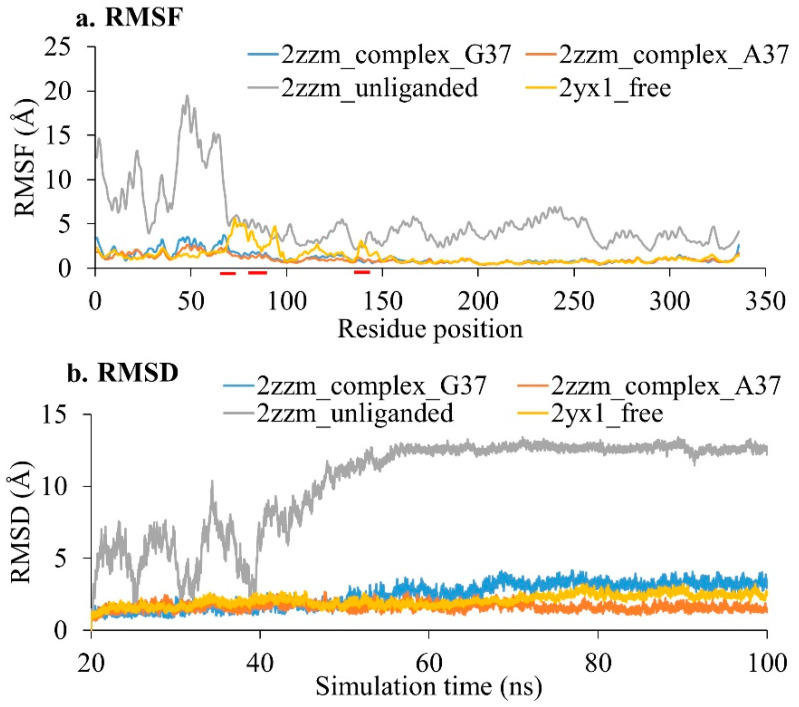
(**a**) Root mean square fluctuation (RMSF) and (**b**) root mean square deviation (RMSD) of a wild-type tRNA methyltransferase complex (2zzm_complex_G37), a complex with G37A tRNA mutation (2zzm_complex_A37), unliganded protein of the complex (2zzm_unliganded), and free protein (2yx1_free). The DOT regions are marked with red colour in the X-axis.

The fluctuations of amino acid residues in the aTrm5 wild-type tRNA complex protein, the aTrm5 G37A mutant tRNA complex, the protein part of aTrm5 complex (unliganded), and the free protein were estimated by root mean square fluctuation (RMSF), and the results are shown in Figure 2a. We observed that the unliganded protein had a very high residue fluctuation, which denotes the poor stability of the protein. Similarly, RMSD calculations of unliganded proteins showed a high variation in the structure for the first 50 ns, as shown in Figure 2b, while the RMSD analysis of all the other systems was stable before 20 ns. The second replication of simulations of wild-type and aTrm5 mutant tRNA complexes showed a root mean square deviation of less than 2 Å (Figure S2).

The aTrm5 wild-type tRNA complex and aTrm5 mutant tRNA complex showed a similar fluctuation for the structures throughout the simulations. While comparing the fluctuations of the free protein with wild-type and mutant complexes, all the DOT regions showed higher fluctuations in both replications of simulations (Figure 2a and Figure S3). These results are expected, since the DOT regions are free to move in the solution in free form and hence have higher fluctuations. Apart from DOT regions, most of the amino acid residues showed a similar fluctuation in the free protein, as well as in wild-type and mutant complexes. Similarly, the comparison of the residue cross correlation map in the wild-type complex and unliganded complex shows that DOT regions have a lower correlated motion (Figure S4). Particularly, DOT3 is shown to have a weaker correlation with nearby residues than DOT1 and DOT2, which suggests that DOT1 and DOT2 are dependent on the movement of nearby residues, which could be due to their intramolecular interactions to fold in the absence of tRNA. The movement of DOT3 region is independent of nearby residues, possibly because of it having less involvement in folding. Interestingly, this observation, along with the fact that the DOT3 region interacts with both the AdoMet ligand and the G37 nucleotide of tRNA, supports their role in the catalysis of aTrm5 enzymes.

#### 3.1.2. Role of DOT Regions in Compactness of Free Protein and RNA Binding in tRNA Methyltransferase Complex

**Figure 3:**
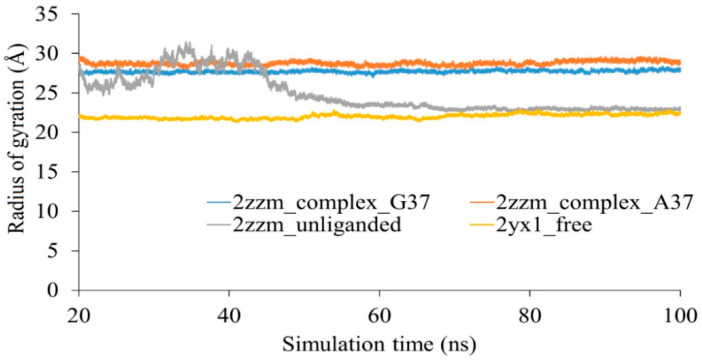
Radius of the gyration of wild-type complex (2zzm_complex_G37), complex with G37A tRNA mutation (2zzm_complex_A37), unliganded complex protein (2zzm_unliganded), and free protein (2yx1_free).

The protein part of aTrm5 is 336 residues long and binds to 84 nucleotides of tRNA. Given the length of the protein and tRNA, the protein can only bind tRNA completely when it has a few extended stretches of structures. These extended stretches present in the complex are disordered in the free protein and have disorder-to-order transition upon binding with tRNA. The DOT regions are observed to be stretched in the protein-tRNA complex (Figure 1a), whereas it is shrunk in the free protein, owing to the greater number of intermolecular interactions (Figure 1b). The radius of gyration calculations, which provide information on the compactness of proteins, showed that the radius of gyration of the protein in protein-tRNA complexes is higher throughout the simulation compared to in the free protein (Figure 3). The free protein has a compact structure, suggesting that DOT regions shrink in three-dimensional space and allow better intramolecular interactions. Interestingly, the unliganded protein is unstable for the first 50 ns, and subsequently attains an equilibrium state similar to that of the free protein. Although the native contacts are not restored in the unliganded protein, the structural compactness is still similar to that of the free protein. The RMSF analysis of the last 40 ns simulation trajectories of unliganded proteins shows that the N-terminal is highly flexible, and the DOT1 and DOT2 regions are dominant in flexibility (Figure S5). Hence, in aTrm5 proteins, DOT1 and DOT2 have major structural rearrangements before the binding of the tRNA molecule. The observations are consistent with the literature, showing that DOT1 and DOT2 are highly flexible in the absence of tRNA and attain a stable structure upon tRNA binding [5,25]. On the other hand, DOT1 and DOT2 are dominated with positively charged residues (DOT1 contains 3 Lys out of 7 residues, and DOT2 contains 3 Lys and 1 Arg out of 10 residues). The DOT3 region is flanked by positively charged residues, such as Arg135, Arg136, Lys137, and Arg147, which bind to the tRNA and contribute to strong binding.

In addition, we analysed the distances among the three domains—i.e., D1 (1–68), D2 (76–175), and D3 (176–336). The distance between D1–D3 domains provides the information on compactness of the protein, which are shown by center of masses (Figure S6) and minimum distances calculations (Figure S7). Free and unliganded proteins are more compact than complexes, and hence have lower D1–D3 distance. The D1–D3 distance of wild-type and mutant complexes range between 40–60 Å, while for the free protein, the distance is stable at about 30 Å. Interestingly, the unliganded protein has an initial center of mass distance of 60Å, which reaches less than 40 Å after 60 ns of the simulations, while the minimum distance reaches less than 5 Å (Figures S6 and S7). The structural comparison of the final frame of unliganded and free proteins shows that DOT1 allows D1 to move freely and establish the interactions between D1 and D3 (Figure S8).

The DOT1 is present in the junction of D1–D2 domains, whereas DOT2 and DOT3 are present in the D2 domain. Hence, the D1–D2 distance can also be used to estimate the change in the shape of DOT1. In wild-type and mutant complexes, the D1–D2 distance is more than 40 Å, while for the free protein the distance is around 30 Å. In the unliganded protein, the initial distance is 40 Å, which reaches 30 Å after 60 ns (Figure S6). This observation, along with the high RMSF of DOT1 and DOT2 regions in unliganded protein, suggests that the flexibility of DOT1 is important for structural changes, which might be necessary for binding to the partner tRNA.

### 3.2. Mutational Study of G37 Nucleotide of the tRNA

tRNA methyltransferase enzymes methylate tRNA at position 37 (wild-type nucleotide is Guanine), which helps to maintain the L-shape structure and increase the efficiency of the translation process. The experimental studies have shown that the methyl group from AdoMet is transferred to G37 nucleotide, which is conserved across three domains of life [25]. In this study, we have modified guanine nucleotide at position 37 with another purine nucleotide—i.e., adenine (G37A)—and analysed the impact of modification in the energy and the interaction pattern. The minimum energy graph shows that in the wild-type complex, the protein is tightly bound to the tRNA. Particularly, a conserved Pro267 is known to interact with G37, which is closer in the wild type than in the mutant complex. The mutation in Pro267 was shown to be associated with a decrease in the catalytic efficiency (k_cat_) of methylation in a steady-state kinetic study [10]. In addition, neighbouring residues are also less distant in the wild-type complex than in the mutant. We noticed that both wild-type and mutant tRNA maintained an L-shape structure throughout the simulations. The L-shape of tRNA is required during the translation process, and hence, further studies are necessary to understand the importance of maintaining the L-shape of tRNA in both wild-type and mutant forms.

**Figure 4:**
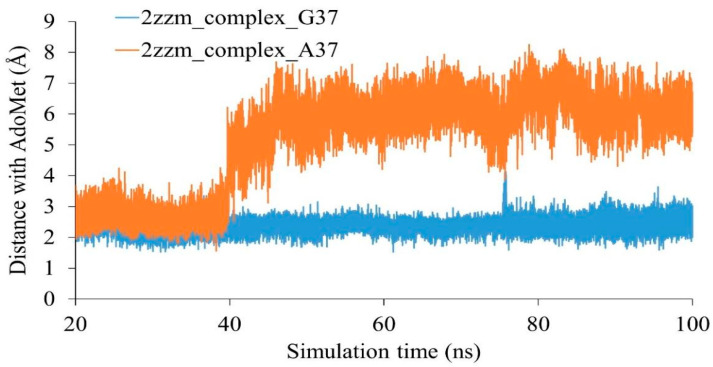
Distance between the 37th nucleotide of tRNA and S-adenosyl methionine in wild-type (2zzm_complex_G37) and mutant tRNA (G37A) complex (2zzm_complex_A37).

During the methylation process, the methyl group of AdoMet is donated to G37. Hence, we calculated the distance between G37 and A37 with the AdoMet ligand in wild-type and mutant complexes (Figure 4). The results showed that in the wild-type complex, the AdoMet ligand interacted with G37 (distance was around 2 Å) throughout the simulations. In the G37A mutant complex, A37 maintained a distance of 2 Å with AdoMet during the first 40 ns and this increased to 6 Å during the last 60 ns.

### 3.3. Structural Aspects of the Interactions between tRNA, Methyltransferase Protein and S-Adenosyl Methionine Ligand

**Figure 5:**
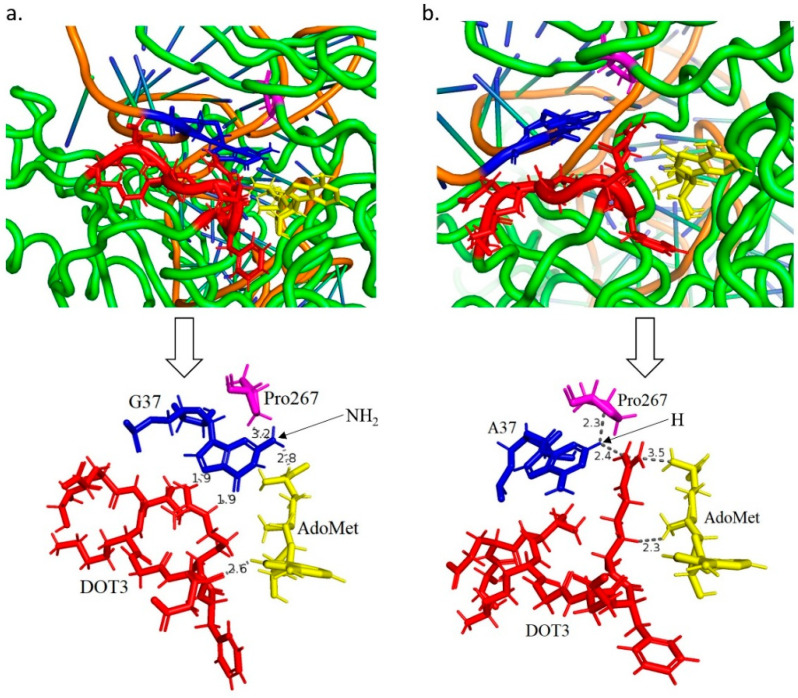
Interaction of DOT3 (red), G37 (blue), AdoMet ligand (yellow), and Pro267 (purple) in (**a**) wild-type and (**b**) G37A complex. The top figures show the positions of DOT3, G37, Pro267, and AdoMet ligand in the overall complex, while the bottom figures only show these regions for better visualisation and interactions analysis. The distance of AdoMet ligand with DOT3, G37, and Pro267 is less than 4 Å. All the distances are in Å.

We investigated the structures of wild-type and mutant (tRNA G37A) aTrm5 proteins, and the structures obtained at 100 ns are presented in Figure 5**.** We observed that the DOT3 region interacts with both the 37th nucleotide of the tRNA as well as the AdoMet ligand. Moreover, the conserved Pro267 residue was also observed to interact with both the G37 nucleotide in the wild-type and A37 nucleotide in the mutant complex. Similarly, Val140 binds to the backbone of the tRNA and Phe144 undergoes π-π interactions with the aromatic ring of AdoMet ligand in both wild-type and mutant complexes. Although the interacting residues mostly bind in a similar manner, the interaction pattern of Arg145 is different. Figure 5a and Figure S9a show that in the wild-type complex, specifically Arg145 interacts with both G37 and AdoMet ligand from sideways to assist the interactions. In the mutant complex, Arg145 is located between A37 and AdoMet ligand, which hinders the interactions (Figure 5b and Figure S9b). Interestingly, in the wild-type complex the methyl group of the ligand was at a distance of 5.1 and 4.8 Å during the 21st and 100th ns of the simulation, while in the mutant complex at the 21st ns the distance was 6.5 Å, and at the 100th ns the interaction was hindered by the Arg145 residue. A possible explanation for this observation could be that the low affinity interaction between A37 nucleotide and AdoMet ligand drove the ligand away, and the gap was filled by Arg145 residue. To substantiate this result, we repeated simulations of wild-type and mutant complexes and observed that Arg145 continued to interact with the AdoMet ligand but shifted away from A37, which suggests a loss of interactions with A37 (Figure S10). This observation is interpreted by comparing the structures of Guanine and Adenine nucleotides; the purine ring of G nucleotide contains NH_2_ and O groups at the 2nd and 6th positions, respectively, while A only contains the NH_2_ group at the 6th position. The NH_2_ group at the 2nd position in the G nucleotide may help to form additional hydrogen bonds and favourable interactions with the G-containing structure. In Figure 5a, the orientation of the 2nd NH_2_ group favours the interaction between G37 nucleotide and the AdoMet ligand, suggesting their possible role in strengthening the interactions. The second replication of the simulation also confirms that the G37 nucleotide in the wild-type structure is closer to the AdoMet ligand than the A37 nucleotide in the mutant structure (Figure S11).

### 3.4. Interaction of DOT Regions with tRNA

**Figure 6:**
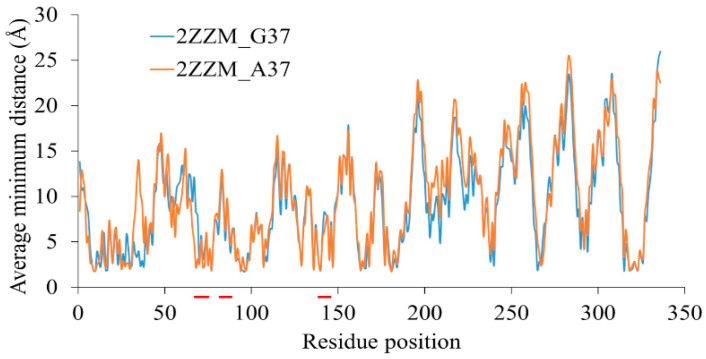
Average minimum distance of wild-type aTrm5 complex (2ZZM_G37) and tRNA G37A mutant aTrm5 complex (2ZZM_A37) for the last 80 ns of simulations. The DOT regions are marked with red colour in the X-axis.

The interactions of DOT regions with tRNA are estimated by average minimum distance calculations, as shown in Figure 6 (results for repetitions are shown in Figure S12). The result shows that all the DOT regions interact with tRNA in both wild-type and mutant complexes. The pattern of interactions is very similar in both the complexes; however, the overall average distance of tRNA and protein is lesser in the wild-type aTrm5 than in the aTrm5 mutant tRNA complex. The final average of the distances also supports the result that residues in the wild-type aTrm5 complex are more tightly packed with the tRNA than the mutant complex.

### 3.5. Energetic Contribution

**Figure 7:**
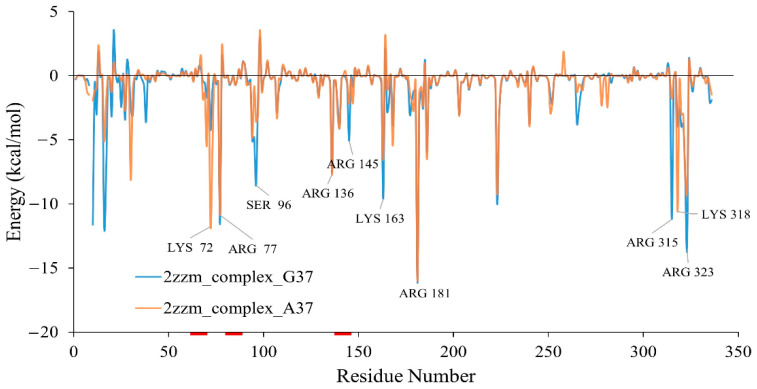
Interaction energy between amino acids of Trm5 and tRNA in wild-type (2zzm_complex_G37) and G37A tRNA mutation complex (2zzm_complex_A37) computed using the MMGBSA method. DOT regions are marked in red colour in the X-axis.

Since the DOT regions favour the interactions, they are expected to contribute favourable interaction energy in the wild-type and aTrm5 mutant tRNA complexes. The energy calculations show that DOT regions and their flanking residue interactions are energetically important for the complex formation, and the results calculated using MMGBSA and MMPBSA are shown in Figure 7 and Figure S13, respectively. Specifically, the residues Lys72, Arg78, Ser96, Arg136, and Arg145 have binding energies of less than −5 kcal/mol in the wild-type aTrm5 complex.

G37 is a conserved nucleotide, and hence the mutation, G37A, at this position is expected to be unfavourable [12,25]. The energy calculation corroborates the hypothesis and shows that most of the residues have weaker energies in the presence of wild-type guanine nucleotide. Specifically, Arg145 has more favourable binding energy in the wild-type than G37A tRNA mutant complex, which is consistent in both MMGBSA and MMPBSA results (Figure 7 and Figure S13). The overall energy calculations of interactions between tRNA and protein residues also confirmed that the energy is more favourable for wild-type than aTrm5 G37A tRNA mutant complex. In addition, the anticodon loop is stabilised by the interactions between tRNA and amino acid residues Pro322 and Arg323. The wild-type (G37) complex has a stronger interaction energy than the aTrm5 mutant tRNA complex.

## 4. Conclusions

Many tRNA-binding proteins have disordered residues, which attain ordered conformations upon binding and are necessary for their functions. tRNA methyltransferase is one such enzyme and is used for the methylation of tRNAs. The archaeal methyltransferase (aTrm5) enzymes contain three disorder-to-order transition (DOT) regions. The DOT regions help these proteins to maintain an extended structure upon binding to the tRNA. In the absence of tRNA, the DOT regions fold back and help the free proteins attain a more compact structure. The radius of gyration calculation shows that the complex is more extended than the free protein. The structural fluctuation analysis and distances between domains show that the DOT regions are crucial in maintaining the structure of the free proteins and complexes. Interestingly, the DOT3 region binds to the G37 nucleotide, as well as the AdoMet ligand, and also influences the binding of ligand with tRNA. For instance, Arg145, which is a part of the DOT3 region, is shown to maintain the interaction between G37 and the AdoMet ligand in the wild-type complex. In the G37A tRNA mutant complex, A37 loses, while Arg145 keeps the interactions with the AdoMet ligand.

In addition, the G37 nucleotide is evolutionarily selected and expected to have strong interactions with aTrm5 proteins [5]. In this study, we have shown that the mutation in this nucleotide is energetically less favourable. Moreover, the wild-type guanine nucleotide maintains a lesser distance with the AdoMet ligand, favouring the transfer of the methyl group, while in the mutant complex the distance between the A37 nucleotide and AdoMet increases to more than 5 Å after 40 ns of simulation. In conclusion, we have shown that the DOT regions are important for the structural stability, and the guanine nucleotide at the 37th position shows better interactions with the protein as well as the AdoMet ligand.

## Data Availability

The data that support the findings of this study are available from the corresponding author upon reasonable request.

## Supplementary Materials

The following are available online at https://www.mdpi.com/2073-4425/12/3/399/s1. Figure S1: Distance of DOT regions with (a) AdoMet ligand and (b) G37 nucleotide in the complex, Figure S2: Root mean square deviation (RMSD) for second replicate of the simulation of wild-type and G37A tRNA mutant methyltransferase complex, Figure S3: Root mean square fluctuation for second replicate of the simulation of wild-type and G37A tRNA mutant methyltransferase complex, Figure S4: Residue cross correlation map of (a) unliganded and (b) complex form of wild-type tRNA methyltransferase, Figure S5: Root mean square fluctuation of unliganded protein for the last 40 ns simulations, Figure S6: Distance between center of mass of D1–D2 (magenta), D1-D3 (orange) and D2–D3 (purple) domains, Figure S7: Minimum distance between D1–D2 (magenta), D1–D3 (orange) and D2-D3 (purple) domains, Figure S8: Superimposed structures of the last frame of unliganded (cyan) and free proteins (pink), Figure S9: Interactions between 37th nucleotide and AdoMet ligand in (a) wild-type and (b) G37A tRNA mutant in 100th ns, Figure S10: Distance between Arg145 and AdoMet ligand in two replicates of simulations (Replicates 1 and 2 are represented by R1 and R2, respectively) in the wild-type and G37A tRNA mutant complexes, Figure S11: Distance between 37th nucleotide of tRNA and S-adenosyl methionine (AdoMet) in the second replicate of simulations in wild-type and mutant (G37A) complex, Figure S12: Average minimum distance of second simulation replicate of wild-type aTrm5 complex and tRNA G37A mutant aTrm5 complex for the last 80 ns of simulations, Figure S13: Energy calculation using MMPBSA method for wild-type and G37A tRNA mutant methyltransferase complex.
