# Supplementary material for: Deciphering the Role of Residues Involved in Disorder-To-Order Transition Regions in Archaeal tRNA Methyltransferase 5
Source: Genes (Basel). 2021 Mar 11;12(3):399. doi: 10.3390/genes12030399 (PMC8000304; doi:10.3390/genes12030399)
Supplement: Supplementary file 1 [file genes-12-00399-s001.pdf]

Deciphering the role of residues involved in disorder-to-order transition regions in archaeal tRNA methyltransferase 5

Ambuj Srivastava[1], Dhanusha Yesudhas[1], Shandar Ahmad[2] and M. Michael Gromiha[1,*]

[1] Department of Biotechnology, Bhupat and Jyoti Mehta School of Biosciences, Indian Institute of Technology Madras, Chennai – 600036, Tamil Nadu, India.

[2] School of Computational and Integrative Sciences, Jawaharlal Nehru University, New Delhi-110067, India.

---

[*]corresponding author

Tel: +914422574138

Fax: +91 44 2257 4102

E-mail: gromiha@iitm.ac.in

Supplementary Information

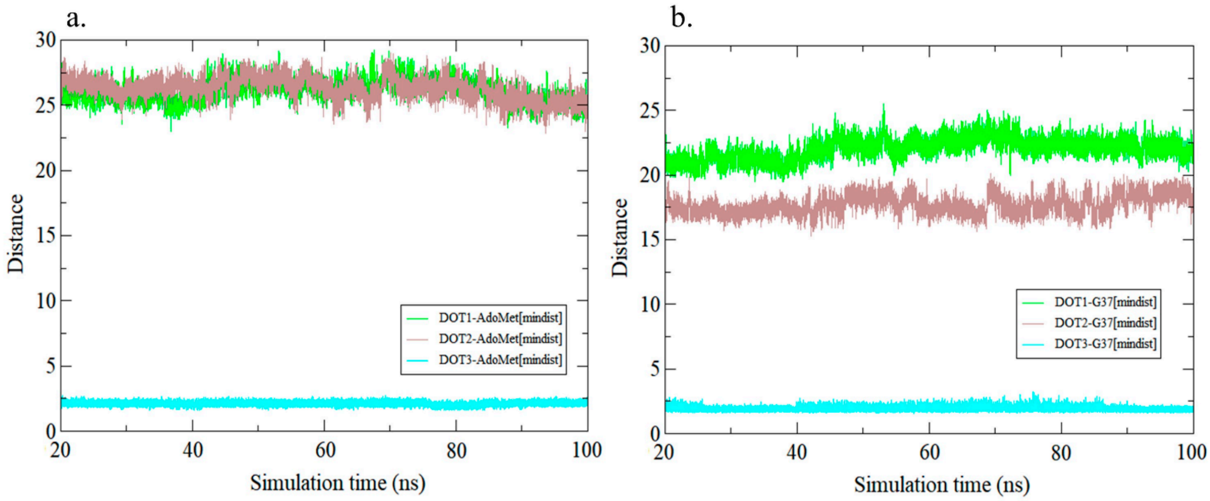

Figure S1: Distance of DOT regions with (a) AdoMet ligand and (b) G37 nucleotide in the complex.

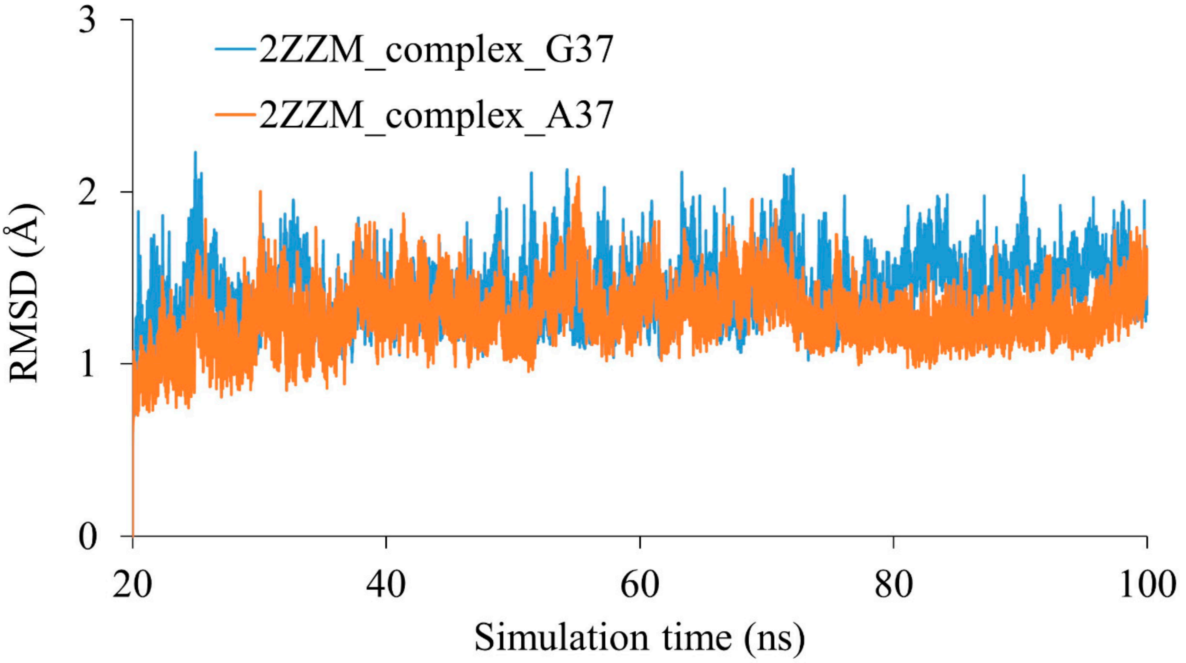

Figure S2: Root mean square deviation (RMSD) for second replicate of the simulation of wild-type and G37A tRNA mutant methyltransferase complex.

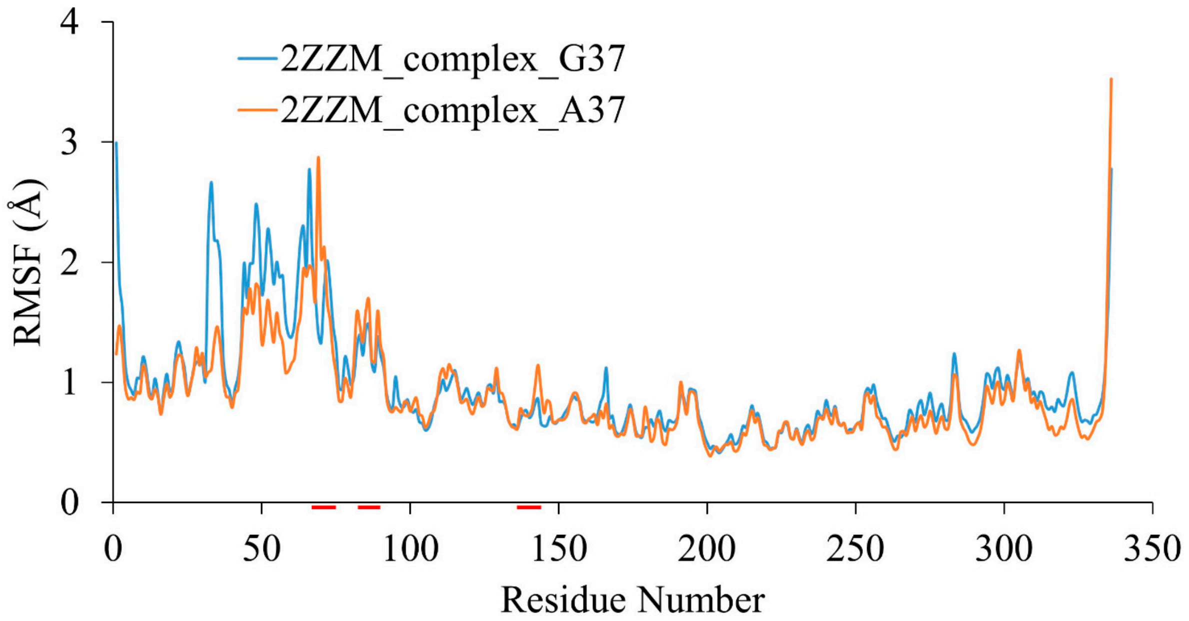

Figure S3: Root mean square fluctuation for second replicate of the simulation of wild-type and G37A tRNA mutant methyltransferase complex. The DOT regions are marked with red color in the X-axis.

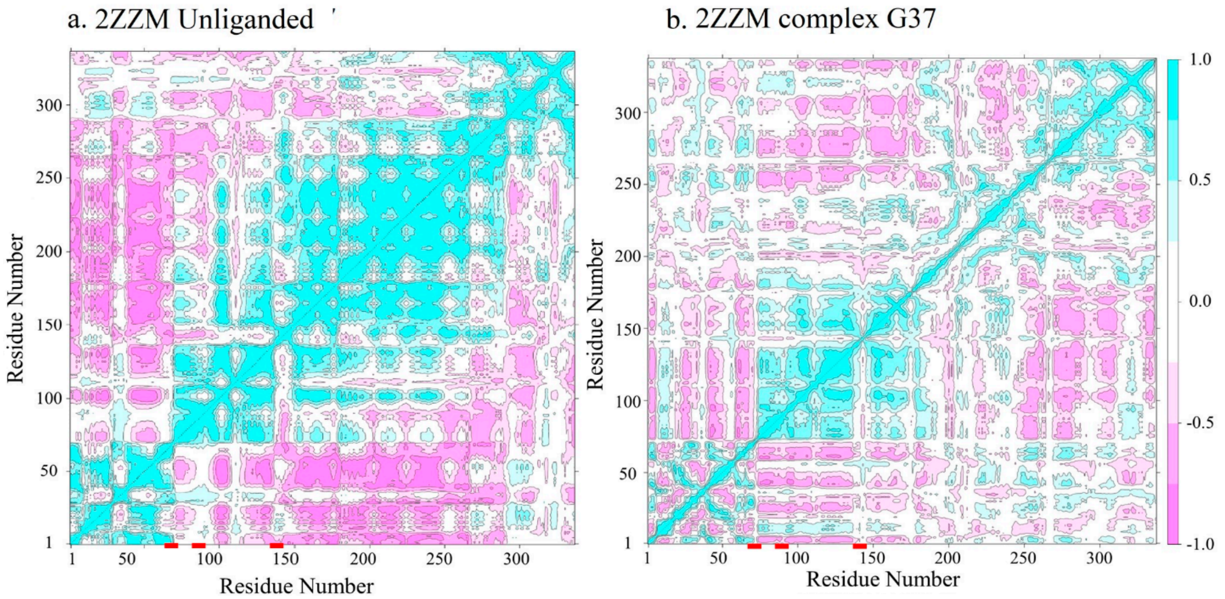

Figure S4: Residue cross correlation map of (a) unliganded and (b) complex form of wild-type tRNA methyltransferase. The DOT regions are marked with red color in the X-axis.

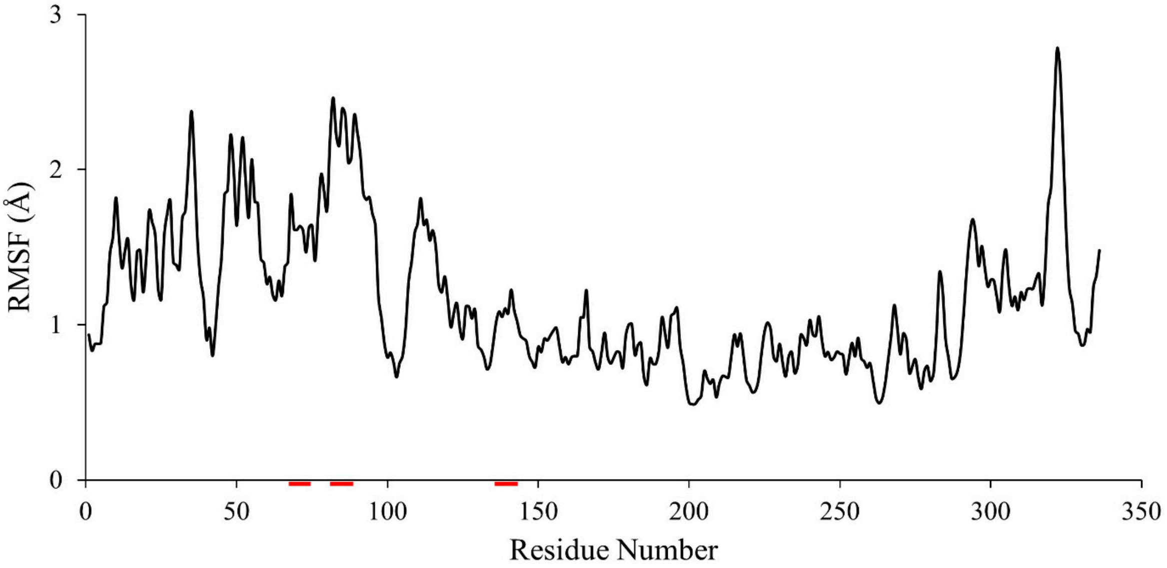

Figure S5: Root mean square fluctuation of unliganded protein for the last 40 ns simulations. The DOT regions are marked with red color in the X-axis.

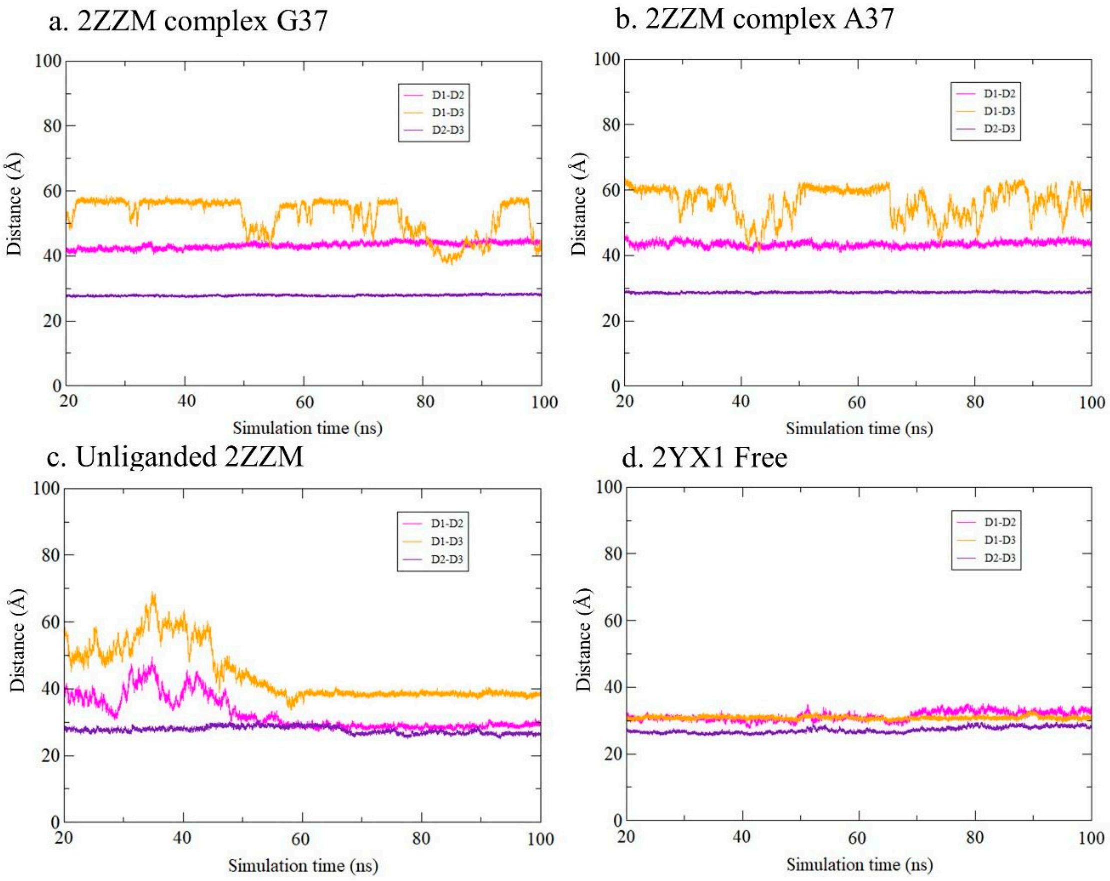

Figure S6: Distance between center of mass of D1-D2 (magenta), D1-D3 (orange) and D2-D3 (purple) domains.

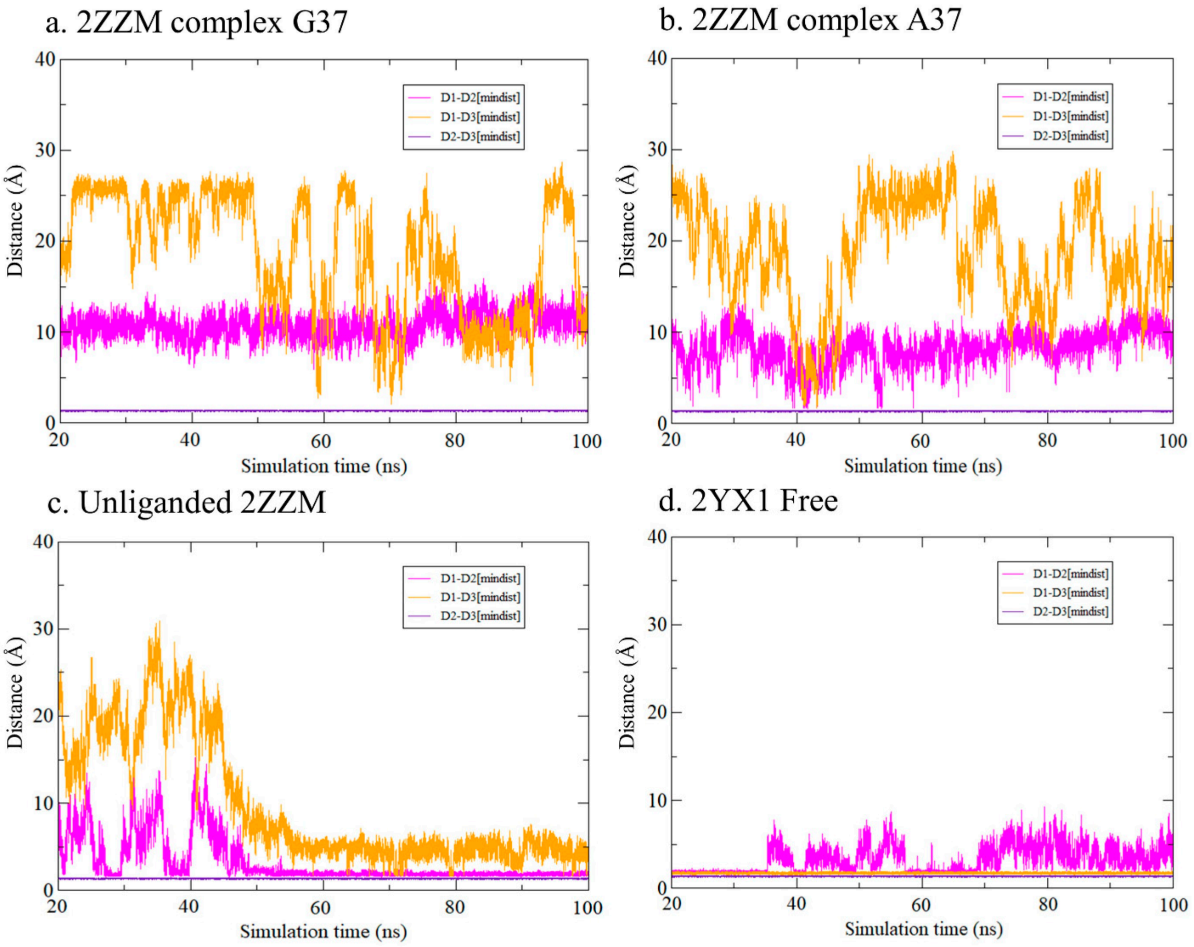

Figure S7: Minimum distance between D1-D2 (magenta), D1-D3 (orange) and D2-D3 (purple) domains.

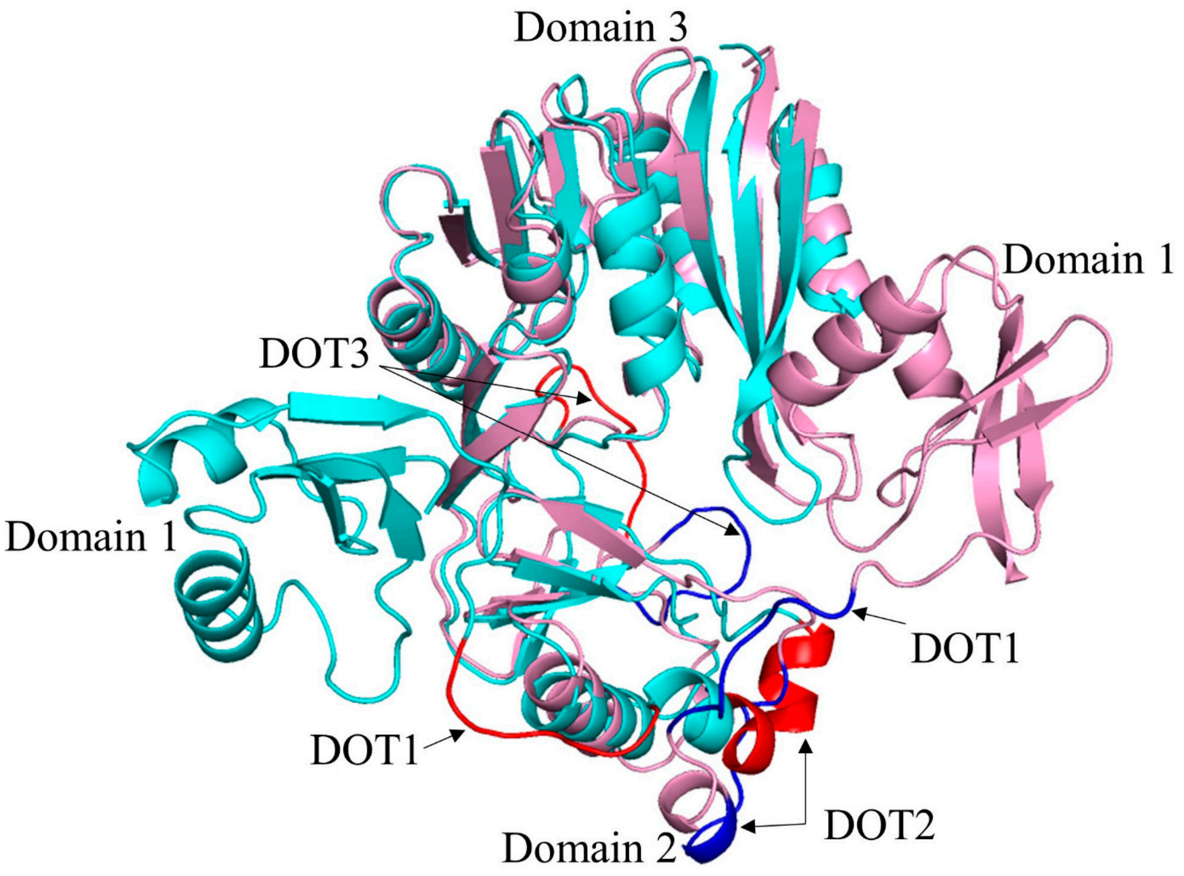

Figure S8: Superimposed structures of the last frame of unliganded (cyan) and free proteins (pink). The DOT regions in unliganded and free proteins are shown in red and blue colors, respectively.

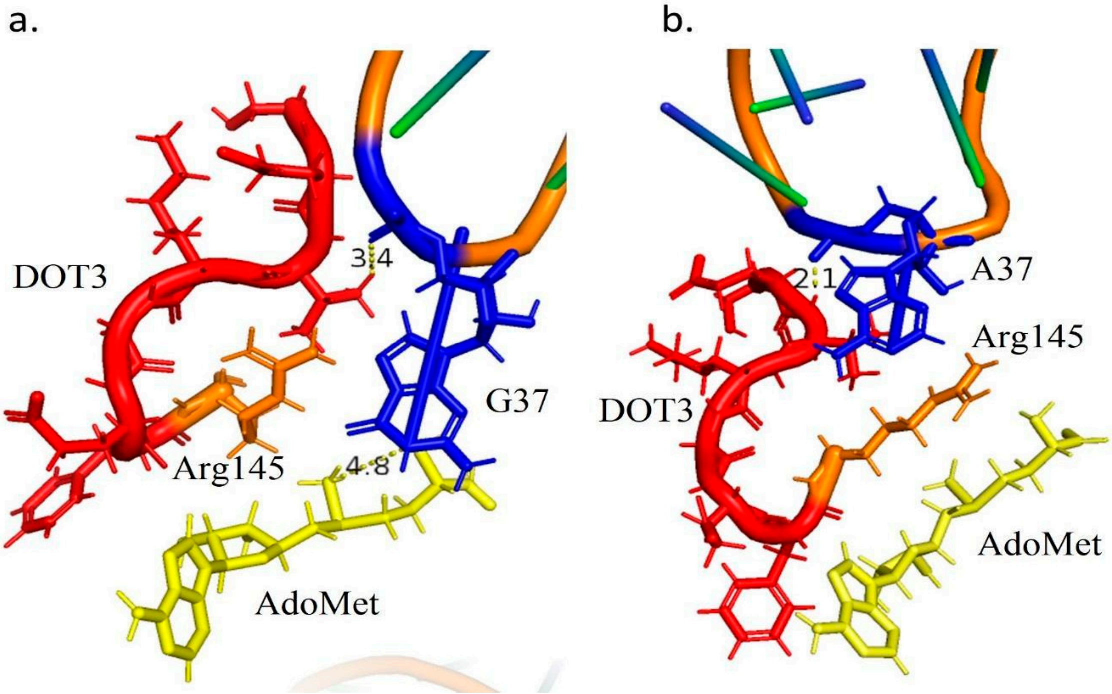

Figure S9: Interactions between 37th nucleotide and AdoMet ligand in (a) wild-type and (b) G37A tRNA mutant in 100th ns. The DOT3 region is shown in red except Arg145 which is orange, G37 and A37 nucleotide is in blue and AdoMet ligand is in yellow color. In wild-type, AdoMet directly interact with G37 while in mutant Arg145 is making a hindrance between Arg145 and A37 nucleotide.

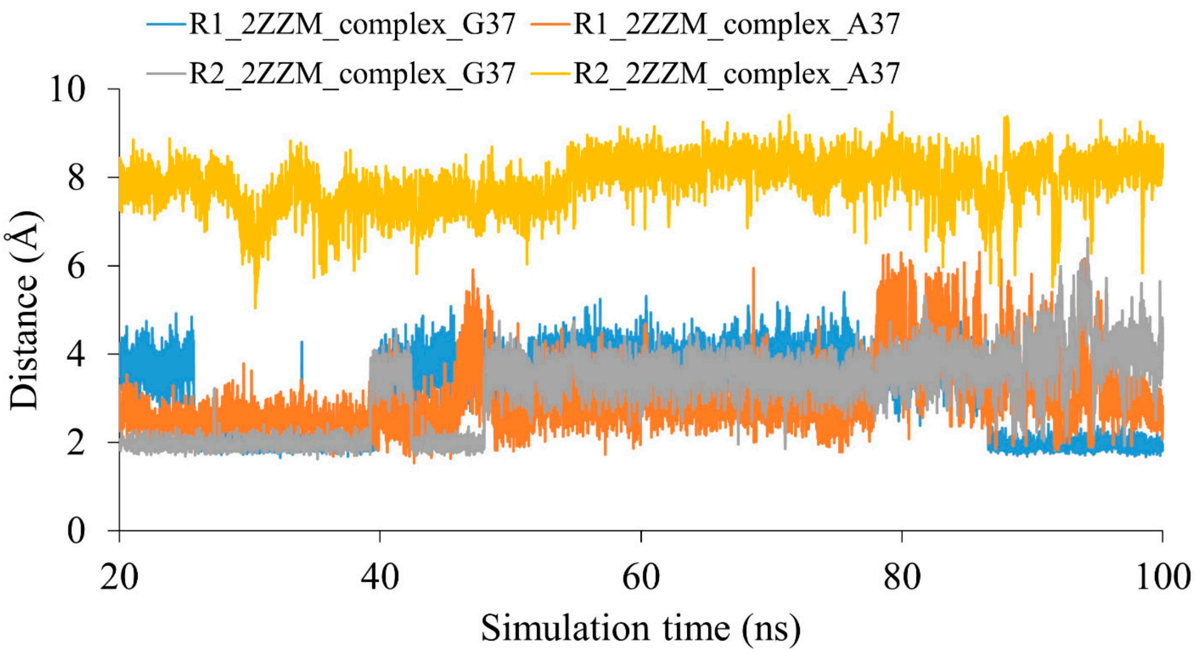

Figure S10: Distance between Arg145 and AdoMet ligand in two replicates of simulations (Replicates 1 and 2 are represented by R1 and R2, respectively) in the wild-type and G37A tRNA mutant complexes.

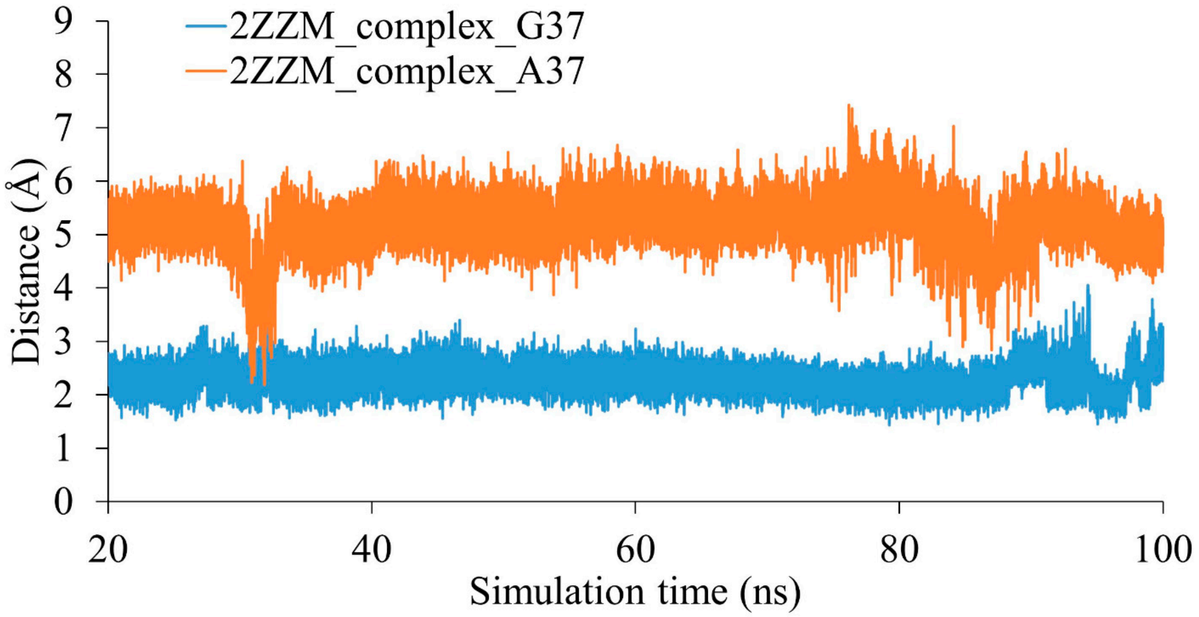

Figure S11: Distance between 37th nucleotide of tRNA and S-adenosyl methionine (AdoMet) in the second replicate of simulations in wild-type and mutant (G37A) complex.

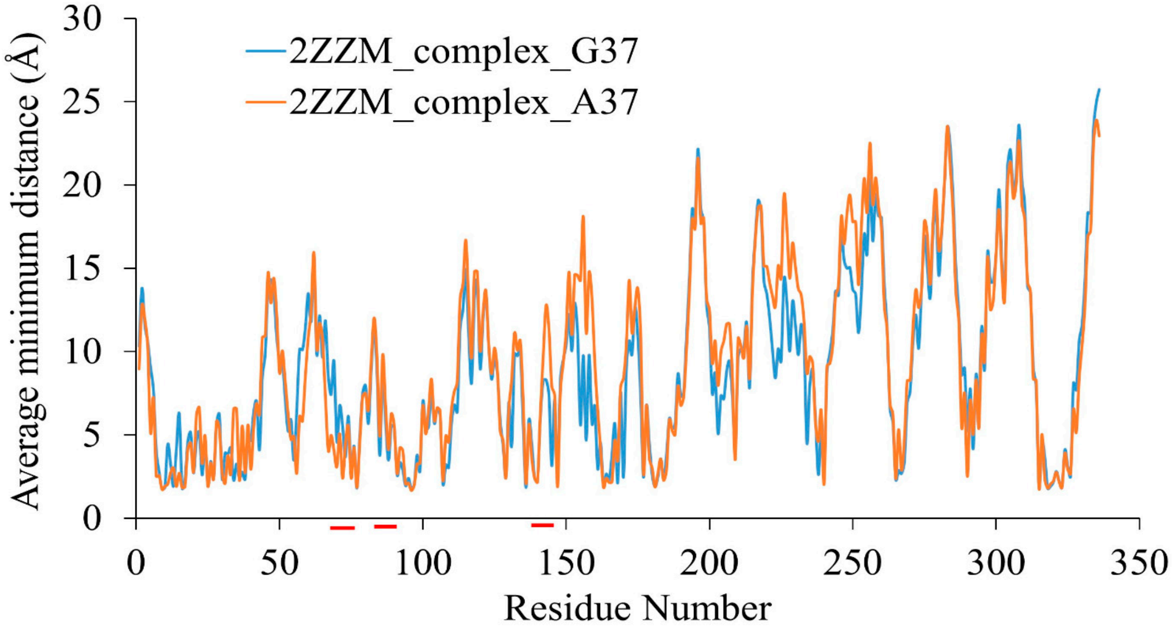

Figure S12: Average minimum distance of second simulation replicate of wild-type aTrm5 complex and tRNA G37A mutant aTrm5 complex for the last 80 ns of simulations. The DOT regions are marked with red color in the X-axis.

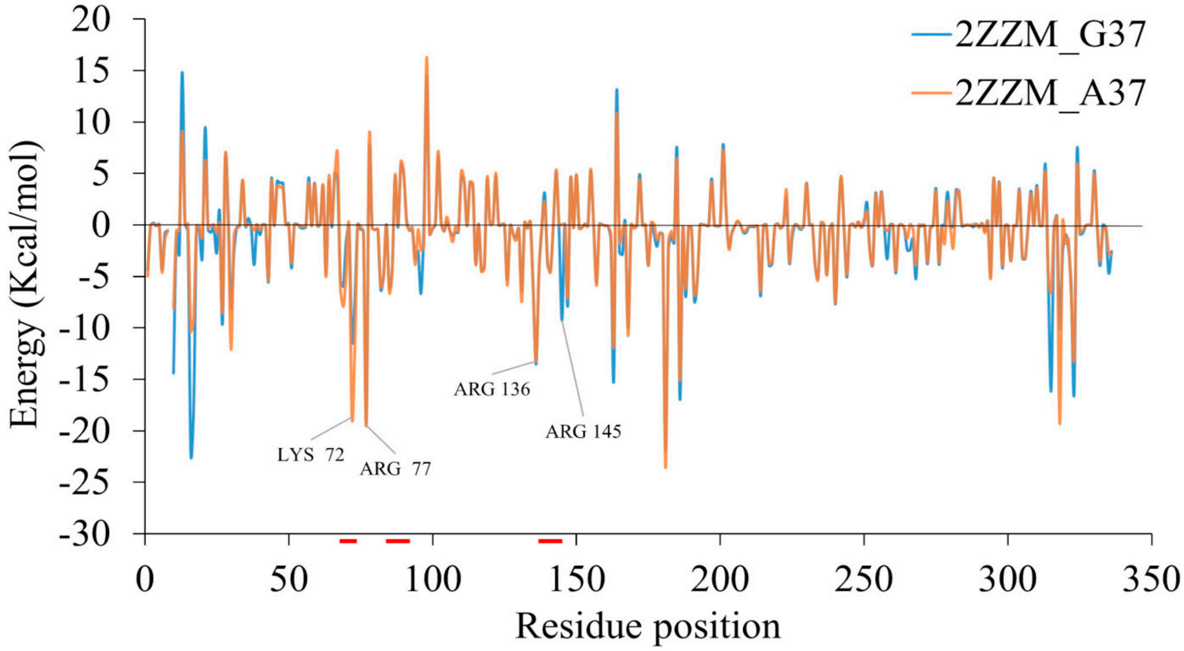

Figure S13: Energy calculation using MMPBSA method for wild-type and G37A tRNA mutant methyltransferase complex. The DOT regions are marked with red color in the X-axis.
